# Supplementary material for: Rhinovirus infection of airway epithelial cells uncovers the non-ciliated subset as a likely driver of genetic risk to childhood-onset asthma
Source: Cell Genom. 2024 Aug 27;4(9):100636. doi: 10.1016/j.xgen.2024.100636 (PMC11480861; doi:10.1016/j.xgen.2024.100636)
Supplement: Document S1. Figures S1–S13 [file mmc1.pdf]

Cell Genomics, Volume *4*

Supplemental information

Rhinovirus infection of airway epithelial cells uncovers the non-ciliated subset as a likely driver of genetic risk to childhood-onset asthma

Sarah Djeddi, Daniela Fernandez-Salinas, George X. Huang, Vitor R.C. Aguiar, Chitrasen Mohanty, Christina Kendziorski, Steven Gazal, Joshua A. Boyce, Carole Ober, James E. Gern, Nora A. Barrett, and Maria Gutierrez-Arcelus

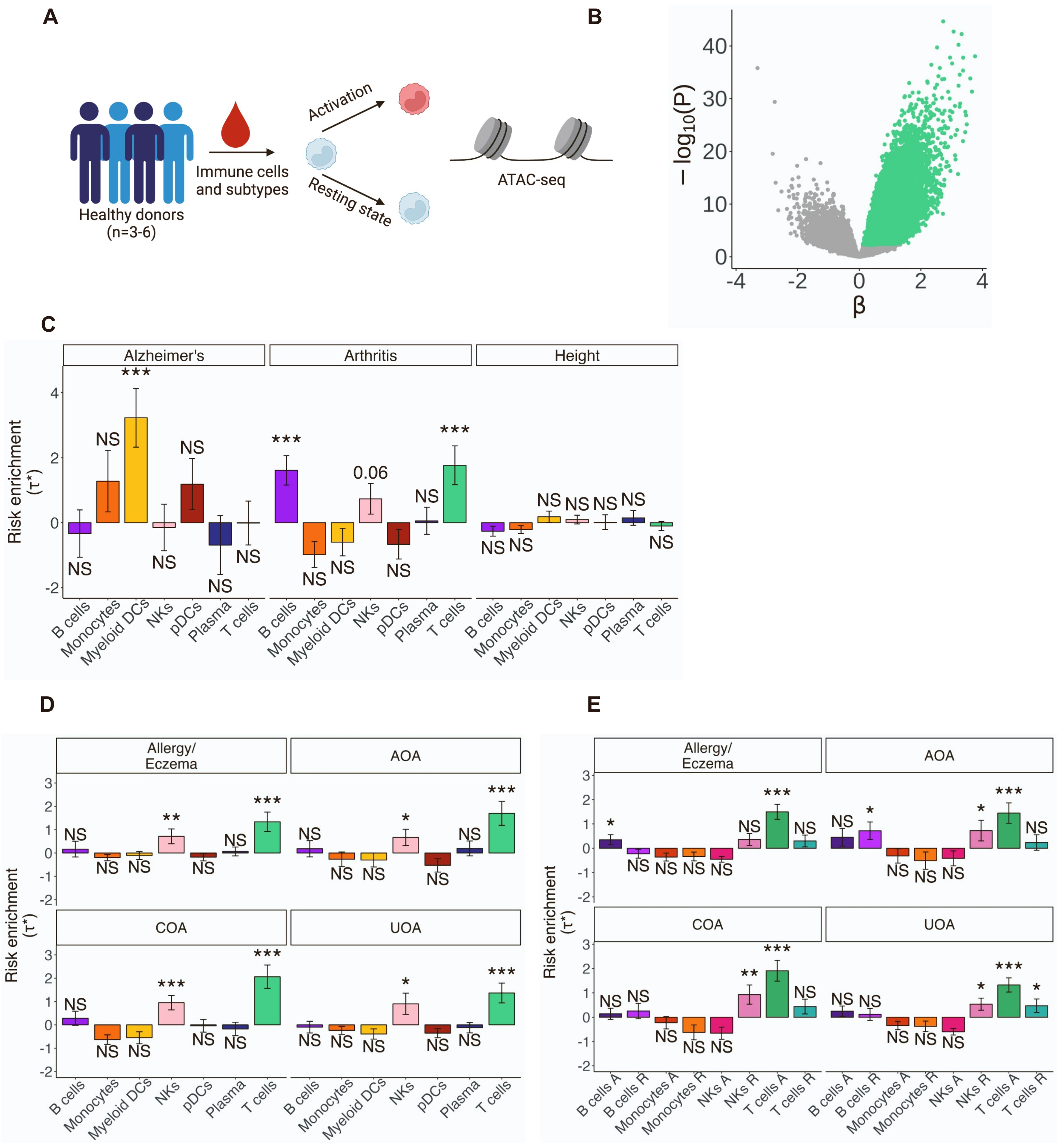

Supplementary Figure 1. Validation of T cells as a relevant cell type for asthma risk using open chromatin data of peripheral blood immune cells. Related to STAR Methods. (A) Experimental design of the *Calderon et al.*[22] ATAC-seq dataset of immune cell types that were activated or not *in vitro*. **(B)** Volcano plot showing differentially accessible peaks between T cells and all other cell types. Peaks with higher accessibility in T cells were selected based on t-statistic and are colored in green. **(C)** Bar plots representing LDSC-SEG heritability enrichment coefficient (τ^*) for each cell type-specific open chromatin annotation for the 3 control traits tested. **(D)** Bar plot representing LDSC-SEG heritability enrichment coefficient (τ^*) for each set of cell-type-specific open chromatin regions for each of the asthma-related traits. **(E)** Bar plots showing LDSC-SEG heritability enrichment coefficient (τ^*) for each of the asthma-related traits in cell-state-specific open chromatin regions of immune cells divided in either resting (light colors) or activated (dark colors) condition. For plasma cells and pDCs there was no in vitro activation performed in the original study. Cell-type-specific results shown for these cell type in panel D depict differential accessible peaks in resting state only. In all bar plots, error bars represent τ^* +/- standard error and asterisks denote significance as *** Bonferroni-adjusted $P < 0.05$, ** FDR 5% , * $P < 0.05$, and NS denotes nonsignificant ($P > 0.05$). AOA: Adult-Onset Asthma, COA: Childhood-Onset Asthma, UOA: Unspecified-Onset Asthma

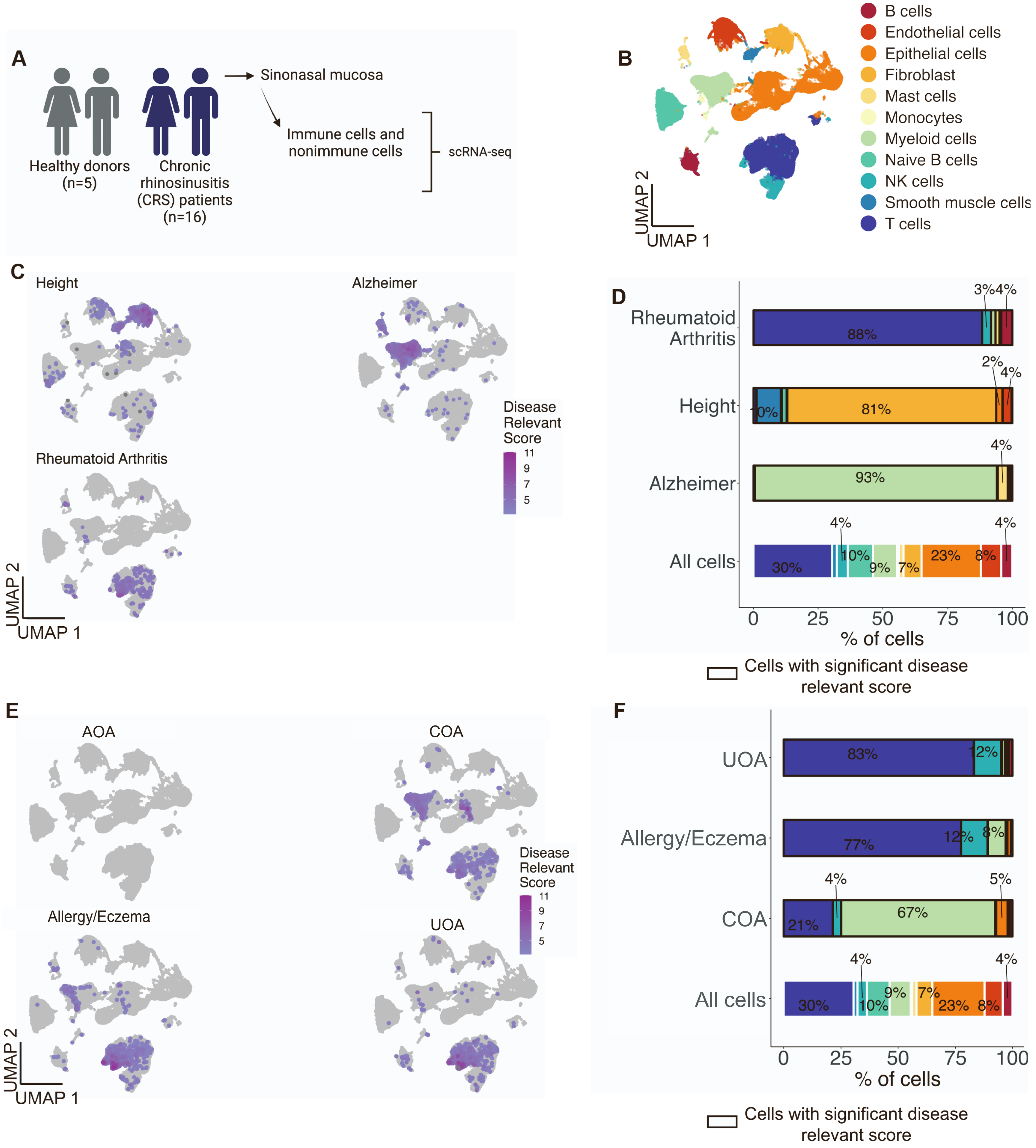

Supplementary Figure 2. Validation of T cells as a relevant cell type for asthma risk using single-cell RNA-seq data. Related to STAR Methods. (A) Experimental design of the *Wang et al.*[39] scRNA-seq dataset of sinonasal mucosa from healthy donors and chronic rhinosinusitis patients (CRS). **(B)** UMAP visualization of the 1,115,856 immune and non-immune cells colored by cell type. **(C)** scDRS results represented on the UMAP for the three control traits tested. Cells with significant disease relevant score at 10% FDR are depicted in purple, with the intensity of the color representing the magnitude of the score. Cells with non-significant score are depicted in gray. **(D)** Bar plot representing the percentage of each cell type in all cells followed by the significant cells at 10% FDR for scDRS in Alzheimer's disease, height, and rheumatoid arthritis.**(E)** scDRS results represented on the UMAP for the four asthma related diseases tested. Cells with significant disease relevant score at 10% FDR are depicted in purple, with the intensity of the color representing the magnitude of the score. Cells with non-significant score are depicted in gray. **(F)** Bar plot representing the percentage of each cell type in all cells in the dataset (bottom bar) and in cells with significant scDRS (10% FDR) for COA, Allergy/Eczema, and UOA. AOA is not represented because no cells were significant. AOA: Adult-Onset Asthma, COA: Childhood-Onset Asthma, UOA: Unspecified-Onset Asthma

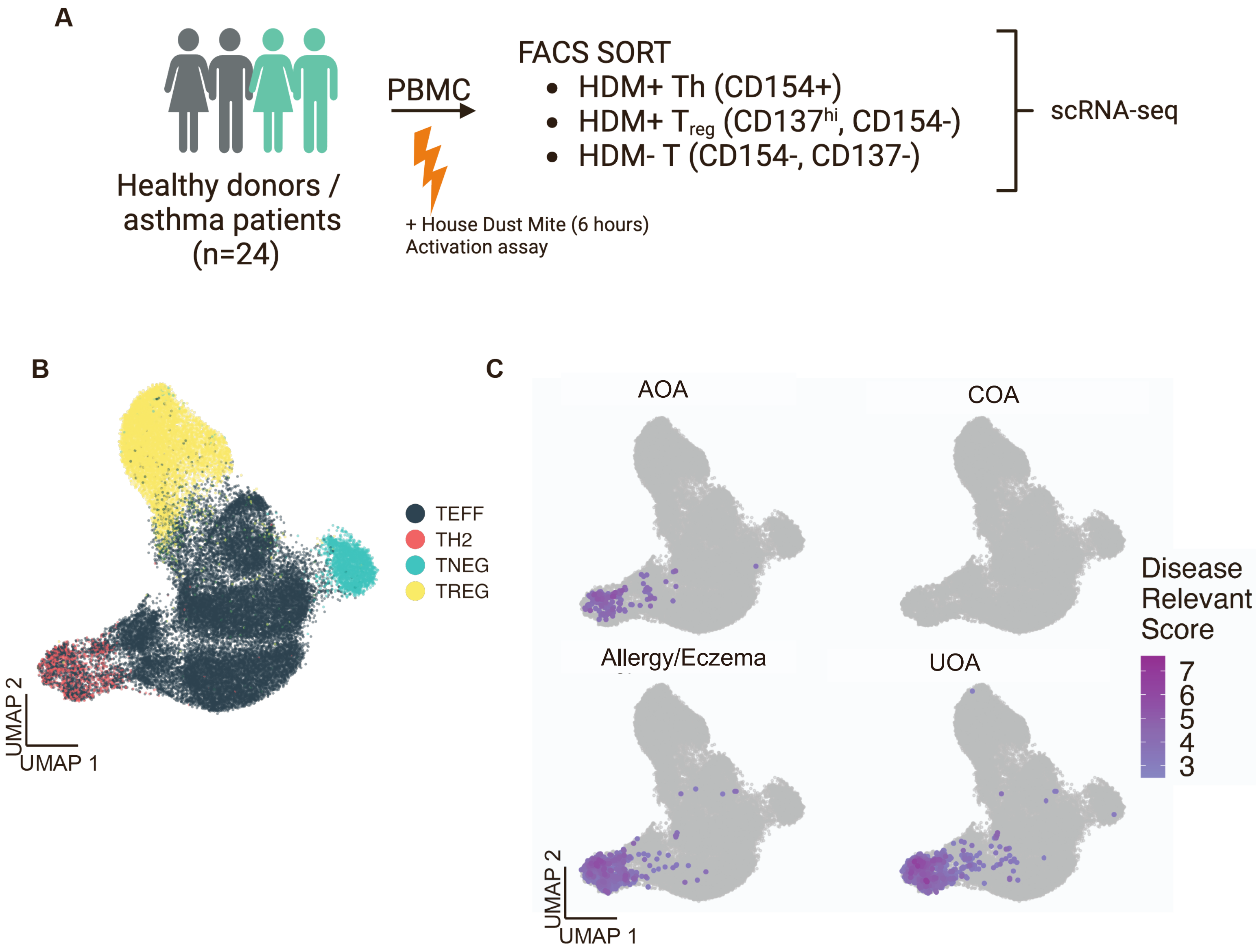

Supplementary Figure 3. Single cell RNA-seq reveals Th2 cells are a relevant T cell subtype for asthma risk. Related to STAR Methods. **(A)** Experimental design of the *Seumois et al.*[40] scRNA-seq dataset consisting of T cells. **(B)** UMAP visualization of the 38,559 T cells colored by subtypes. **(C)** scDRS results represented on the UMAP for the 4 asthma-related diseases tested. Cells with significant disease relevant score at 10% FDR are depicted in purple, with the intensity of the color representing the magnitude of the score. Cells with non-significant score are depicted in gray. AOA: Adult-Onset Asthma, COA: Childhood-Onset Asthma, UOA: Unspecified-Onset Asthma

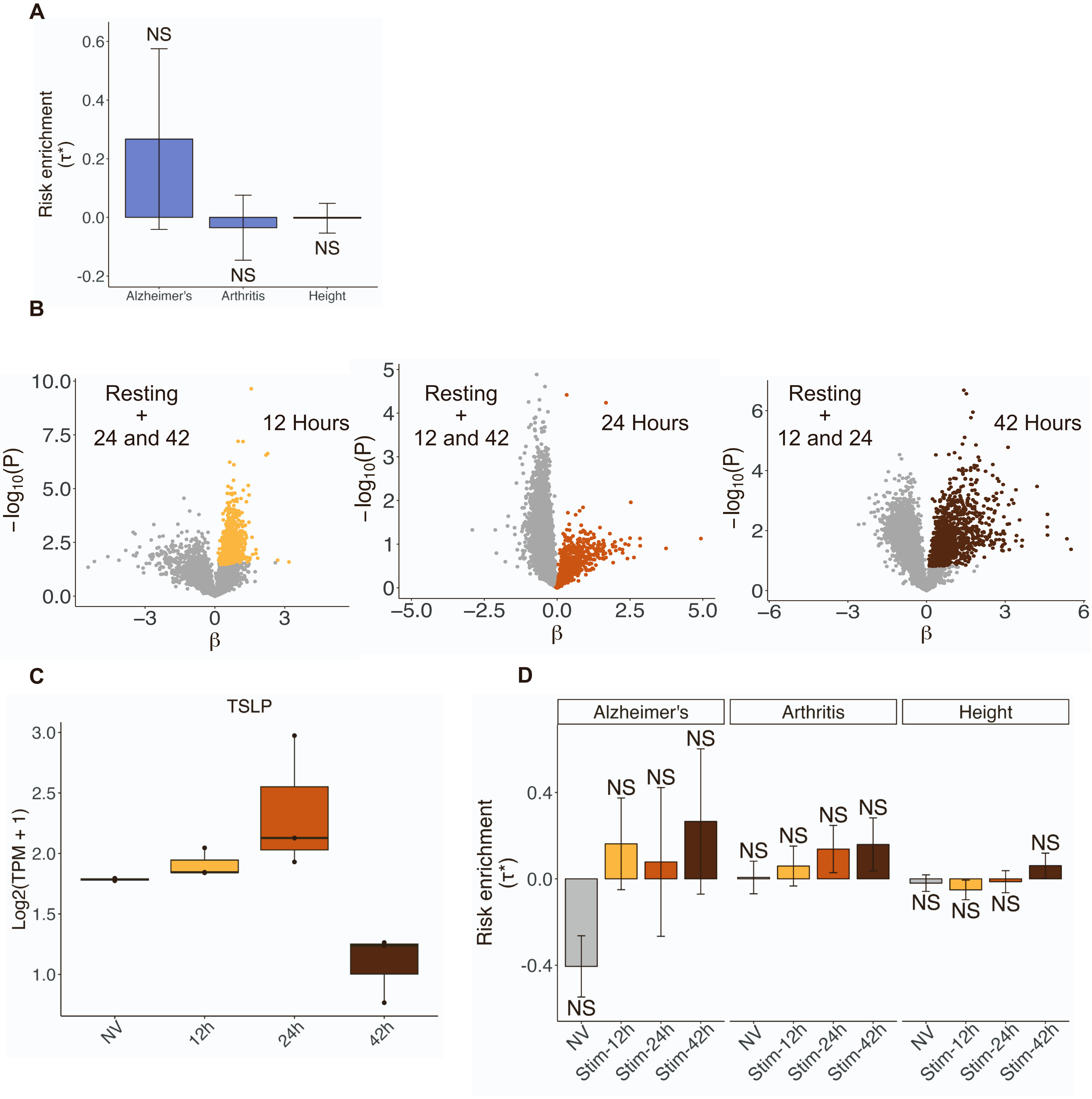

Supplementary Figure 4. Related to Figure 1. Control traits do not show significant heritability enrichment in rhinovirus upregulated genes in bronchial epithelial cells from healthy donors. (A) Bar plots representing LDSC-SEG heritability enrichment coefficient (τ*) for the 3 control traits, using upregulated genes with RV from healthy donors of the *Helling et al.*[41] dataset. NS denotes nonsignificant ($P > 0.05$). **(B)** Volcano plots showing differentially expressed genes at each time point compared to all others in RV-infected airway epithelial cells from healthy donors of the *Basnet et al.*[42] dataset. **(C)**Box plot depicting gene expression levels for TSLP in epithelial cells from the *Basnet et al.*[42] dataset; boxes are colored by time point.**(D)** Bar plot representing LDSC-SEG heritability enrichment coefficient (τ*) for time point-specific genes in each of the control traits tested. NS denotes nonsignificant ($P > 0.05$). In all bar plots, error bars represent τ* +/- standard error.

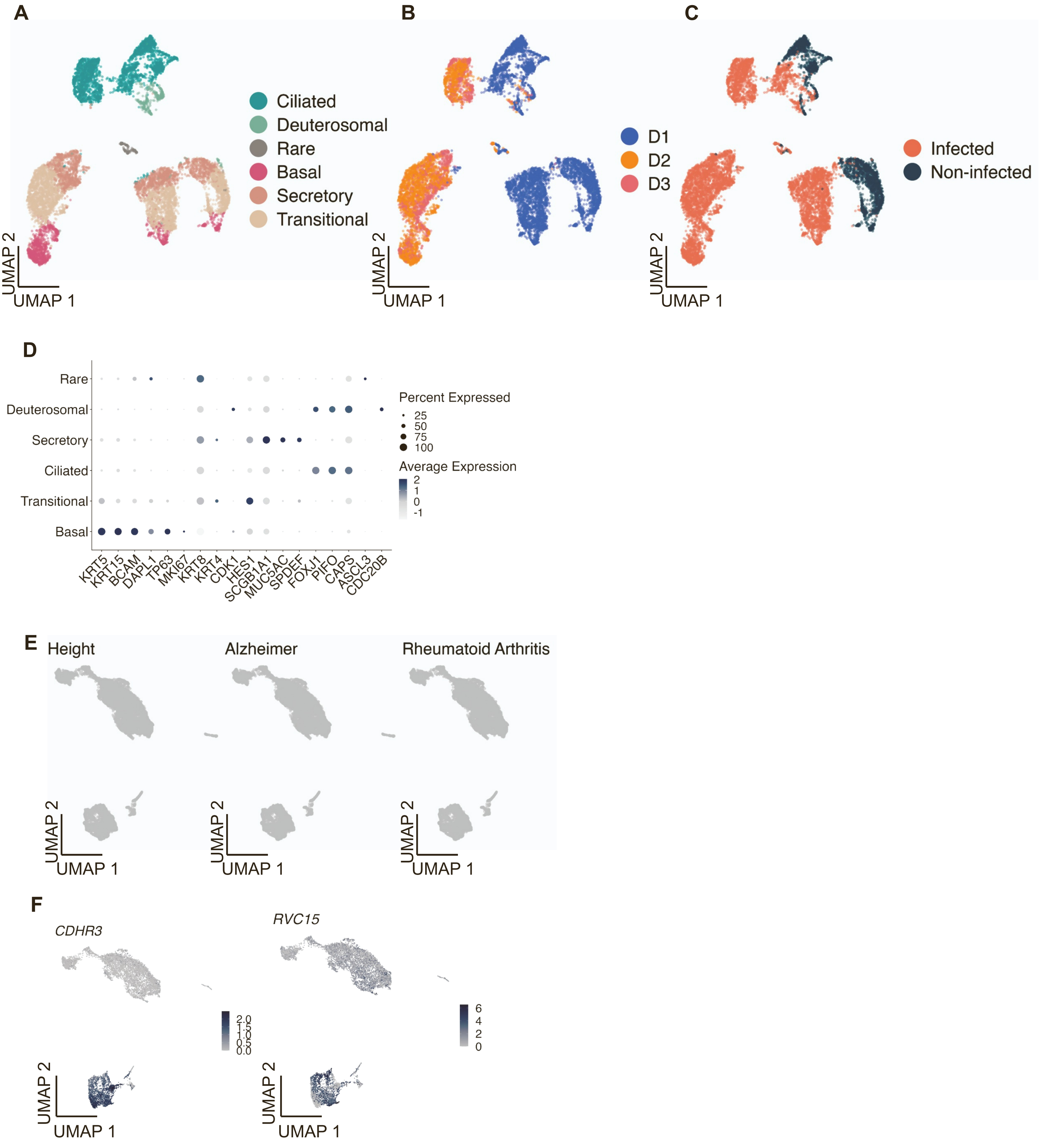

Supplementary Figure 5. Single cell RNA-seq of airway epithelial cells infected with rhinovirus C15. Related to Figure 2. UMAP visualization of the 10,721 epithelial cells of *Basnet et al.*[42] colored by **(A)** cell type, **(B)** donor, **(C)** infection status (Infected / Non-infected). **(D)** Dot plot representing the normalized average expression and the percent of cells expressing a given gene for epithelial cell markers. **(E)** scDRS results represented on the UMAP for the 3 control traits tested. Nonsignificant cells with are depicted in gray. **(F)** Harmonized UMAP representing *CDHR3*, *RVC15* normalized expression levels.

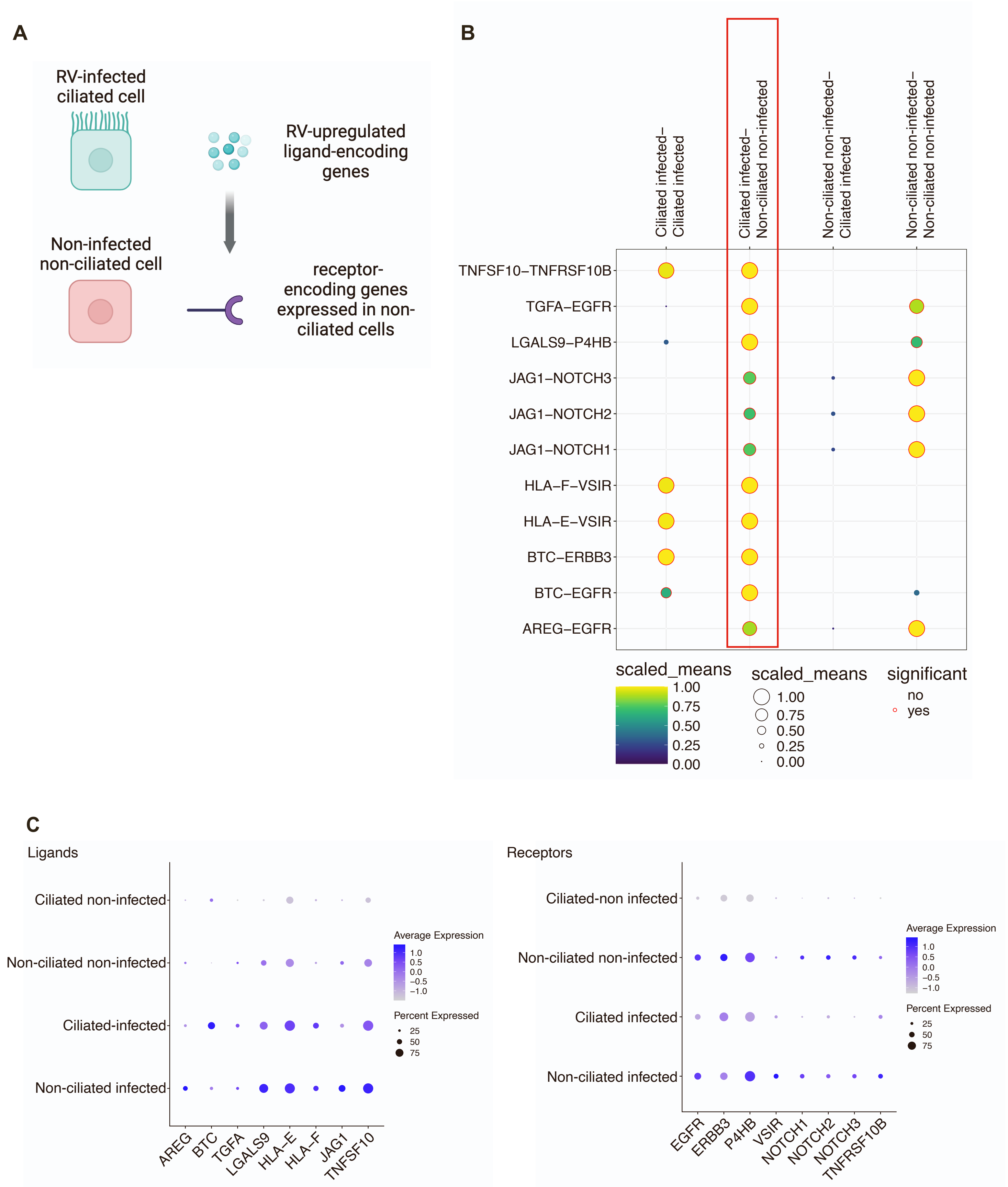

Supplementary Figure 6. Potential ligand-receptor interactions mediating communication between ciliated and non-ciliated cells during rhinovirus infection. Related to Figure 2. (A) Scheme illustrating the rationale for our cellphoneDB analysis: selection of ligand encoding genes upregulated in ciliated cells upon rhinovirus infection, and selection of receptor encoding genes expressed in non-ciliated non-infected cells. **(B)** Dot plot showing cellphoneDB results filtered for significant (permutation p value < 0.05) ligand-receptor interaction between RV-infected ciliated cells and non-ciliated non-infected cells and requiring that the ligand is significantly upregulated upon rhinovirus infection (interacting cell subsets of interest highlighted by a red box). The color and the size of the dot indicate the scaled mean expression levels of ligand and receptor. **(C)** Dot plot representing the normalized average expression and the percent of cells expressing genes that are ligands (left) or receptors (right).

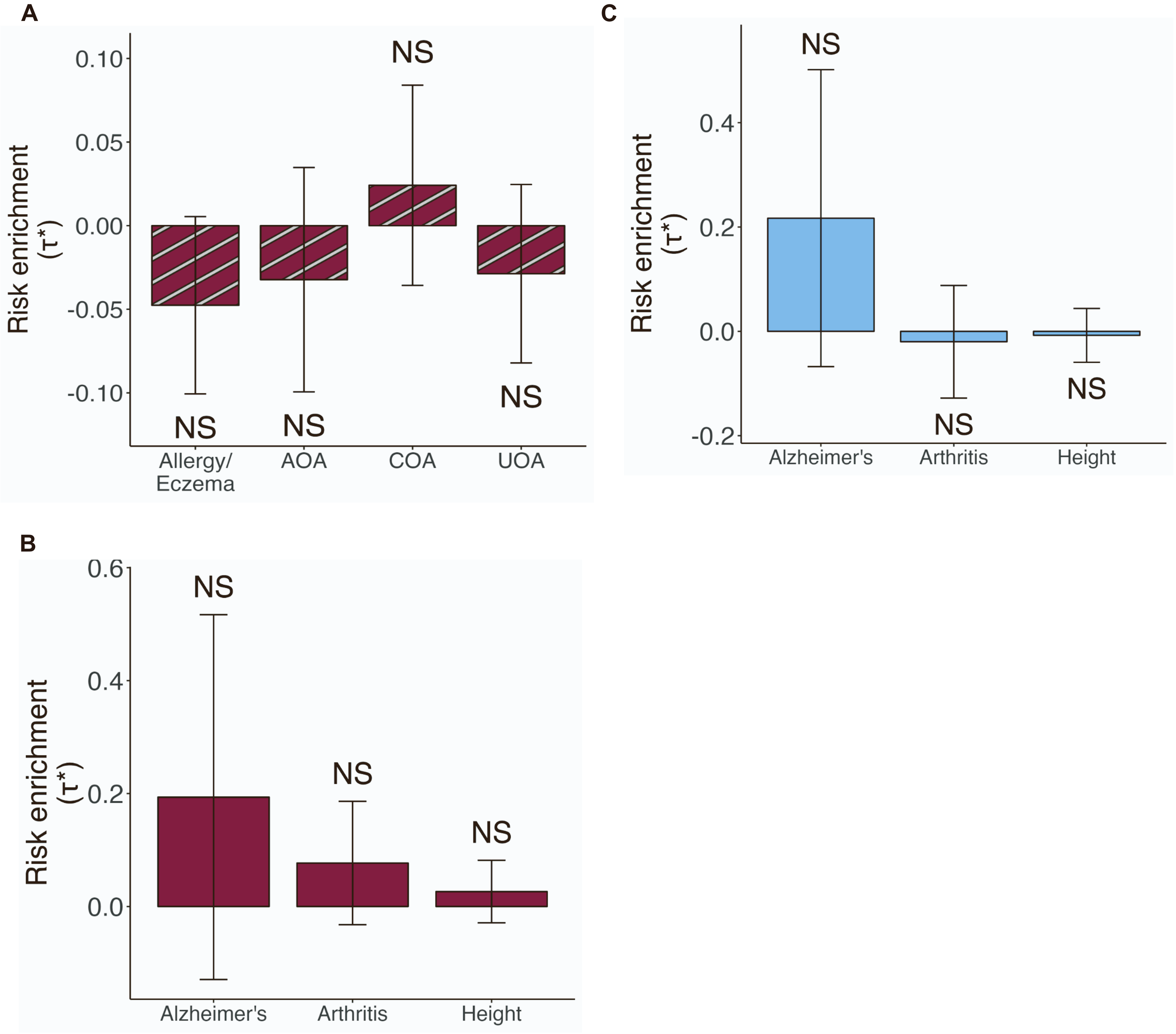

Supplementary Figure 7. Control traits do not show significant heritability enrichment in rhinovirus upregulated genes in bronchial epithelial cells from asthma patients. Related to Figure 3. (A) Bar plot showing LDSC-SEG heritability enrichment coefficient (τ*) across traits for genes downregulated upon RVC-15 infection in airway epithelial cells from patients of *Helling et al.*[41] dataset. **(B)** Bar plots representing LDSC-SEG heritability enrichment coefficient (τ*) for the 3 control traits tested using upregulated genes after RV-infection **(C)** Using genes upregulated in asthmatics when compared to healthy individuals. NS denotes nonsignificant ($P > 0.05$). In all bar plots, error bars represent τ* +/- standard error. AOA: Adult-Onset Asthma, COA: Childhood-Onset Asthma, UOA: Unspecified-Onset Asthma

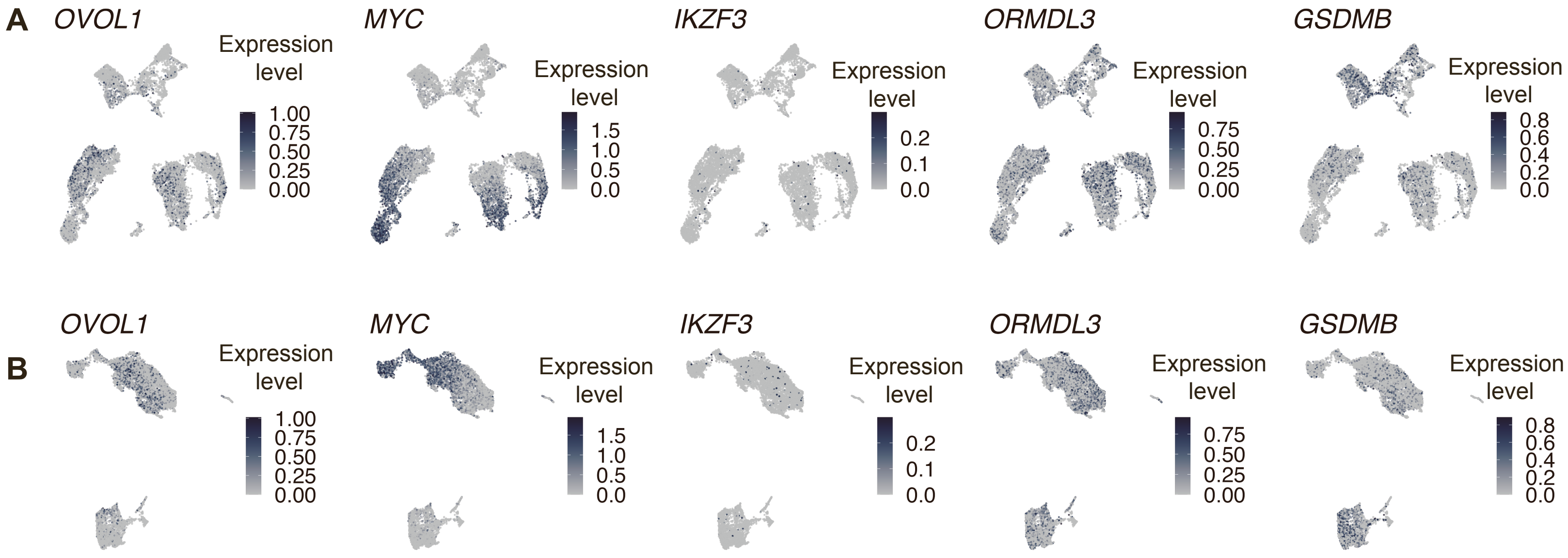

Supplementary Figure 8. Level of expression of genes of interest in single cell RNA-seq data of airway epithelial cells infected with rhinovirus C15. Related to Figure 4. Normalized expression of genes of interest represented on the non-harmonized UMAP **(A)** and the harmonized UMAP **(B)**.

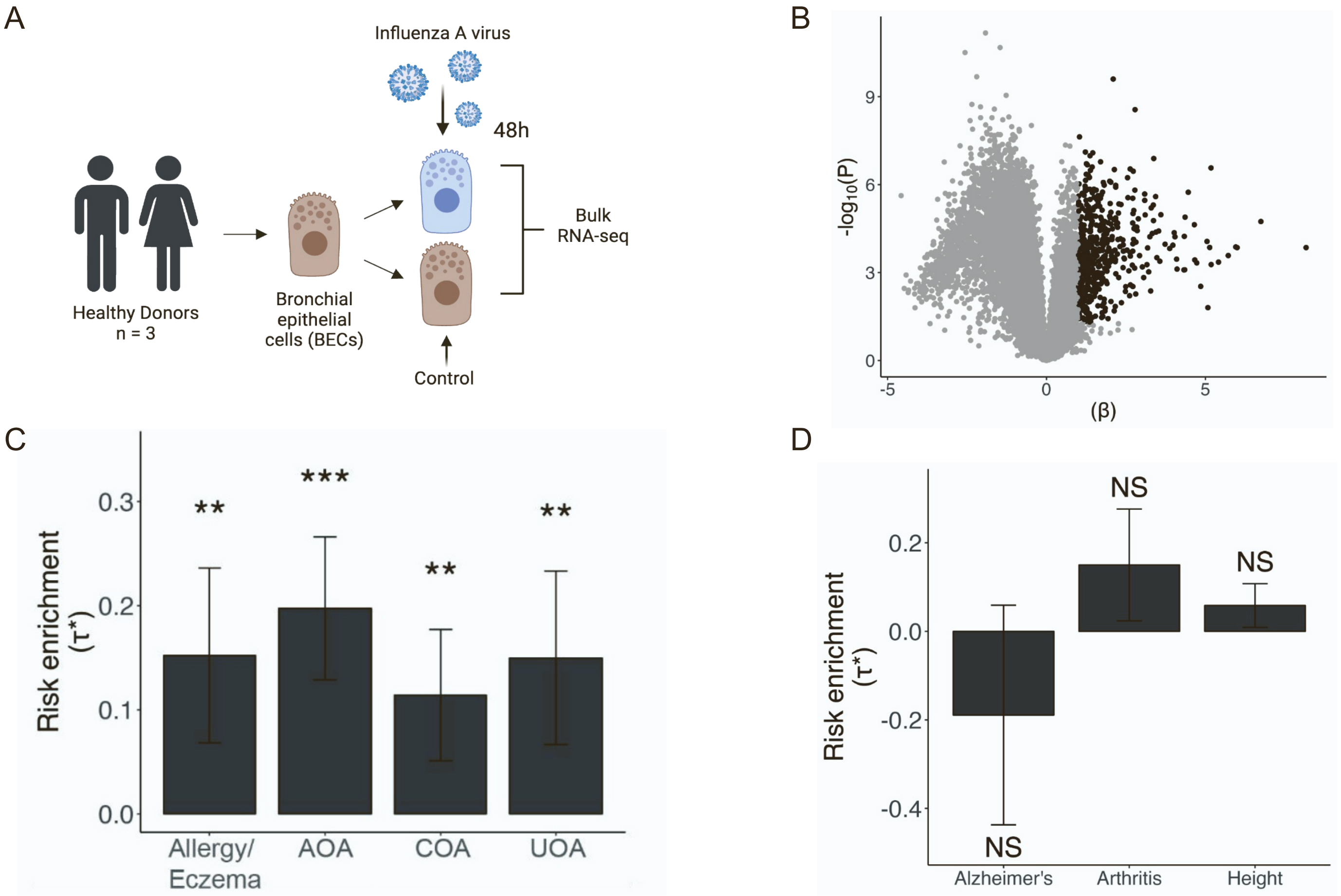

Supplementary Figure 9. Results for Influenza dataset. Related to STAR methods. **(A)** Experimental design of *Tao et al.*[52] bulk RNA-seq dataset. **(B)** Volcano plot showing differentially expressed genes between influenza infection and control. Genes upregulated upon influenza infection were selected based on t-statistic and are colored in black. **(C)** Bar plot representing LDSC-SEG results for upregulated genes after influenza A infection. Error bars represent τ* +/- standard error. Asterisk denotes significance as * $P < 0.05$. **(D)** Bar plots representing LDSC-SEG heritability enrichment coefficient (τ*) the 3 control traits tested. Error bars represent τ* +/- standard error. NS denotes nonsignificant ($P > 0.05$).

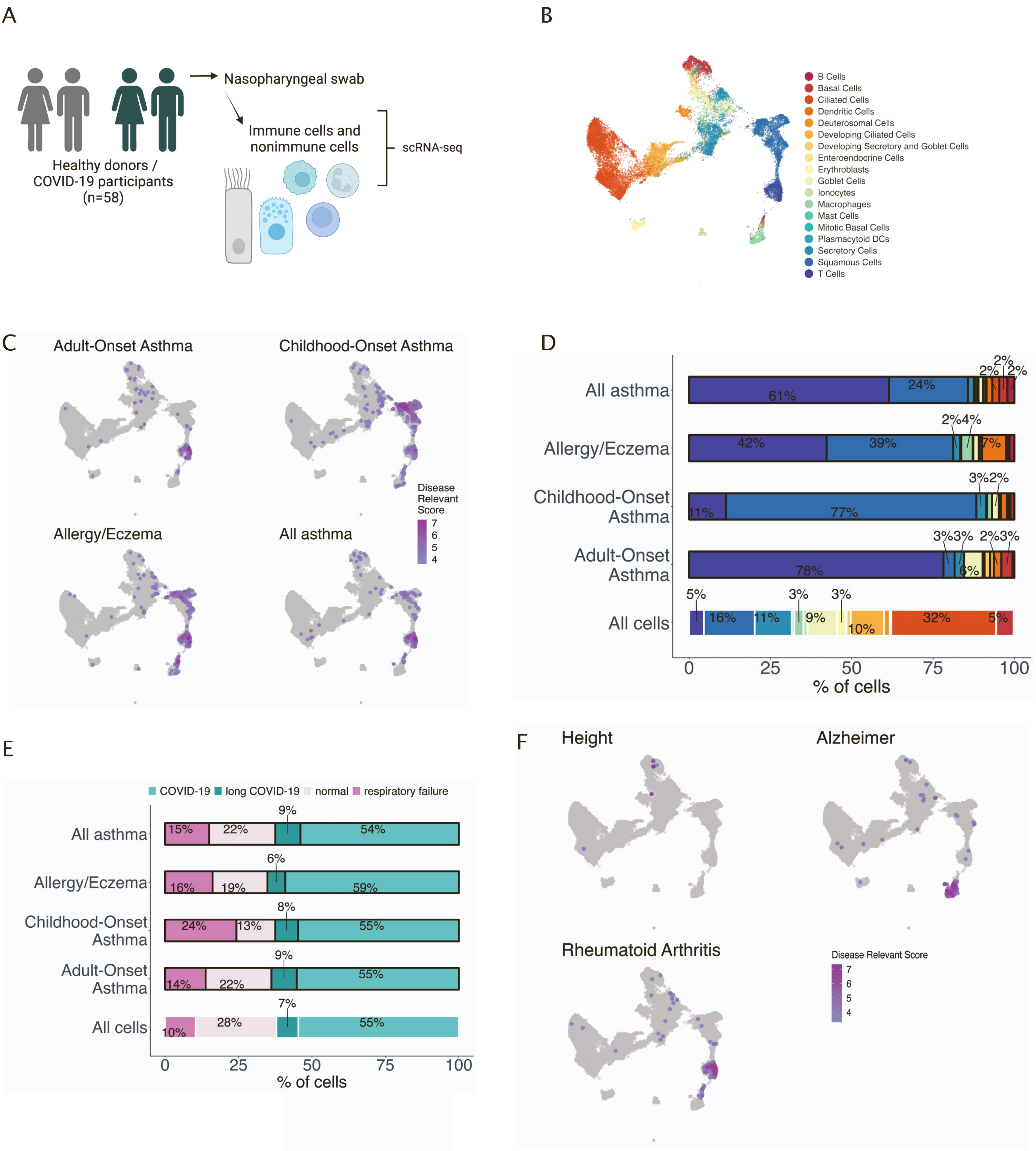

Supplementary Figure 10. Results for COVID-19 dataset. Related to STAR methods. (A) Experimental design of Ziegler et al.[53] single-cell RNA-seq dataset. (B) UMAP visualization of the 32,588 cells colored by cell type. (C) scDRS results represented on the UMAP for the 3 control traits tested. Cells with significant disease relevant score at 10% FDR are depicted in purple, with the intensity of the color representing the magnitude of the score. Cells with non-significant score are depicted in gray. (D) Bar plot representing the percentage of each cell type in all cells followed by the significant cells at 10% FDR for scDRS in AOA, COA, Allergy/Eczema and UOA. (E) Bar plot representing the percentage of cells coming from patients grouped by COVID categories (COVID-19, long COVID-19, respiratory failure) or from healthy donors (normal), for all cells in the dataset (bottom bar), or for cells with significant disease relevant score at 10% FDR for AOA, COA, Allergy/Eczema, UOA. (F) scDRS results represented on the UMAP for the 3 control traits tested. Cells with significant disease relevant score at 10% FDR are depic-ted in purple, with the intensity of the color representing the magni-tude of the score. Cells with non-significant score are depicted in gray. AOA: Adult-Onset Asthma, COA: Childhood-Onset Asthma, UOA: Unspecified-Onset Asthma

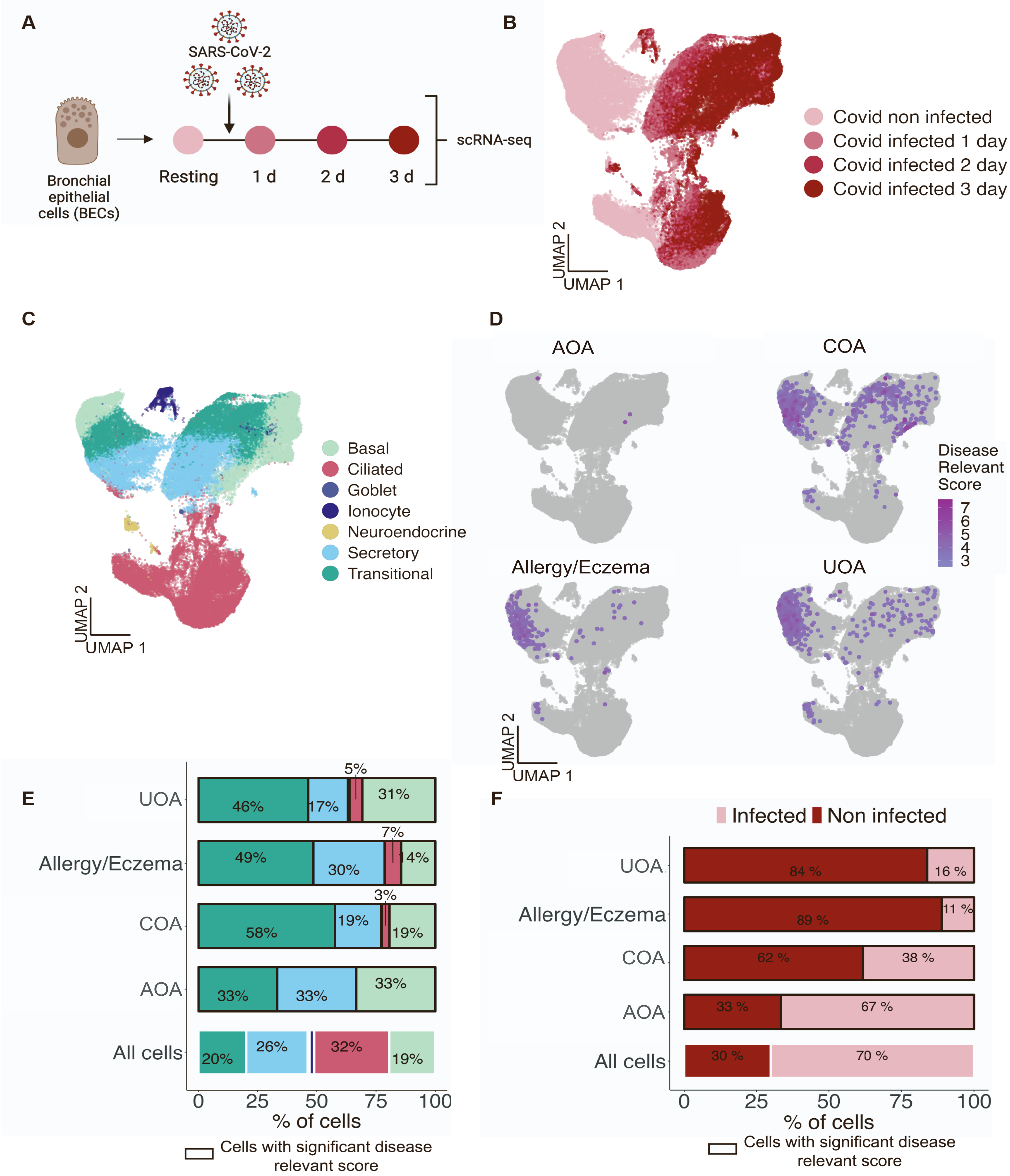

Supplementary Figure 11. scRNA-seq analysis of bronchial epithelial cells infected or not with SARS-CoV-2. Related to STAR methods. (A) Experimental design of the *Ravindra et al.*[54] scRNA-seq dataset of bronchial epithelial cells infected with SARS-CoV-2 or not, from one healthy donor. UMAP visualization of the 74,088 cells colored by (B) days after virus infection and **(C)** by cell type. **(D)** scDRS results represented on the UMAP for the 3 control traits tested. Cells with significant disease relevant score at 10% FDR are depicted in purple, with the intensity of the color representing the magnitude of the score. Cells with non-significant score are depicted in gray. **(E)** Bar plot representing the percentage of each cell type in all cells in the dataset (bottom bar) followed by the scDRS significant cells at 10% FDR for AOA, COA, Allergy/Eczema and UOA. **(F)** Bar plot representing the percentage of cells classified by SARS-CoV-2 infection status in the full dataset (bottom bar), followed by the scDRS significant cells at 10% FDR for AOA, COA, Allergy/Eczema and UOA AOA: Adult-Onset Asthma, COA: Childhood-Onset Asthma, UOA: Unspecified-Onset Asthma

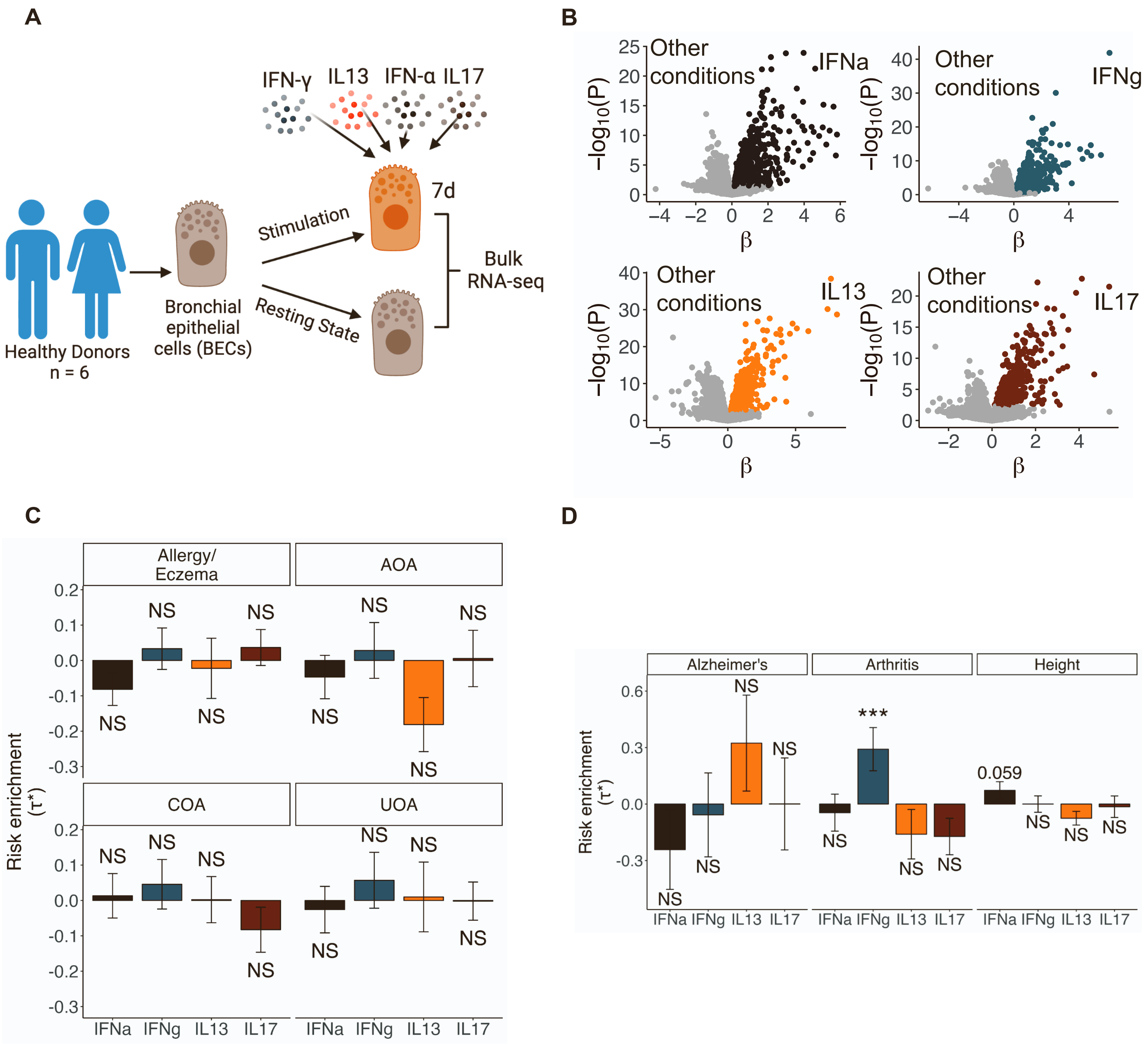

Supplementary Figure 12. Analysis of genes induced by cytokines (IFNγ, IFNα, IL-13, IL-17) in airway epithelial cells. Related to STAR methods. (A) Experimental design of the Koh et al.[62] bulk RNA-seq dataset of BECs from healthy donors that were stimulated or not with different cytokines (IFNγ, IFNα, IL-13, IL-17). **(B)** Volcano plots showing differentially expressed genes for each stimuli (IFNα, IFNγ, IL-13 and IL-17) compared to all others in bronchial epithelial cells colored in black, teal, orange and brown respectively. **(C)** Bar plot representing LDSC-SEG heritability enrichment coefficient (τ*) for genes up-regulated in each stimuli against all others, for each of the asthma-related diseases. Error bars represent τ* +/- standard error. NS denotes nonsignificant ($P > 0.05$). **(D)** Bar plot representing LDSC-SEG enrichment for stimulus-specific expression for each of the 3 control traits. Error bars represent τ* +/- standard error. Asterisk denotes significance as * $P < 0.05$ and NS denotes nonsignificant ($P > 0.05$). AOA: Adult-Onset Asthma, COA: Childhood-Onset Asthma, UOA: Unspecified-Onset Asthma

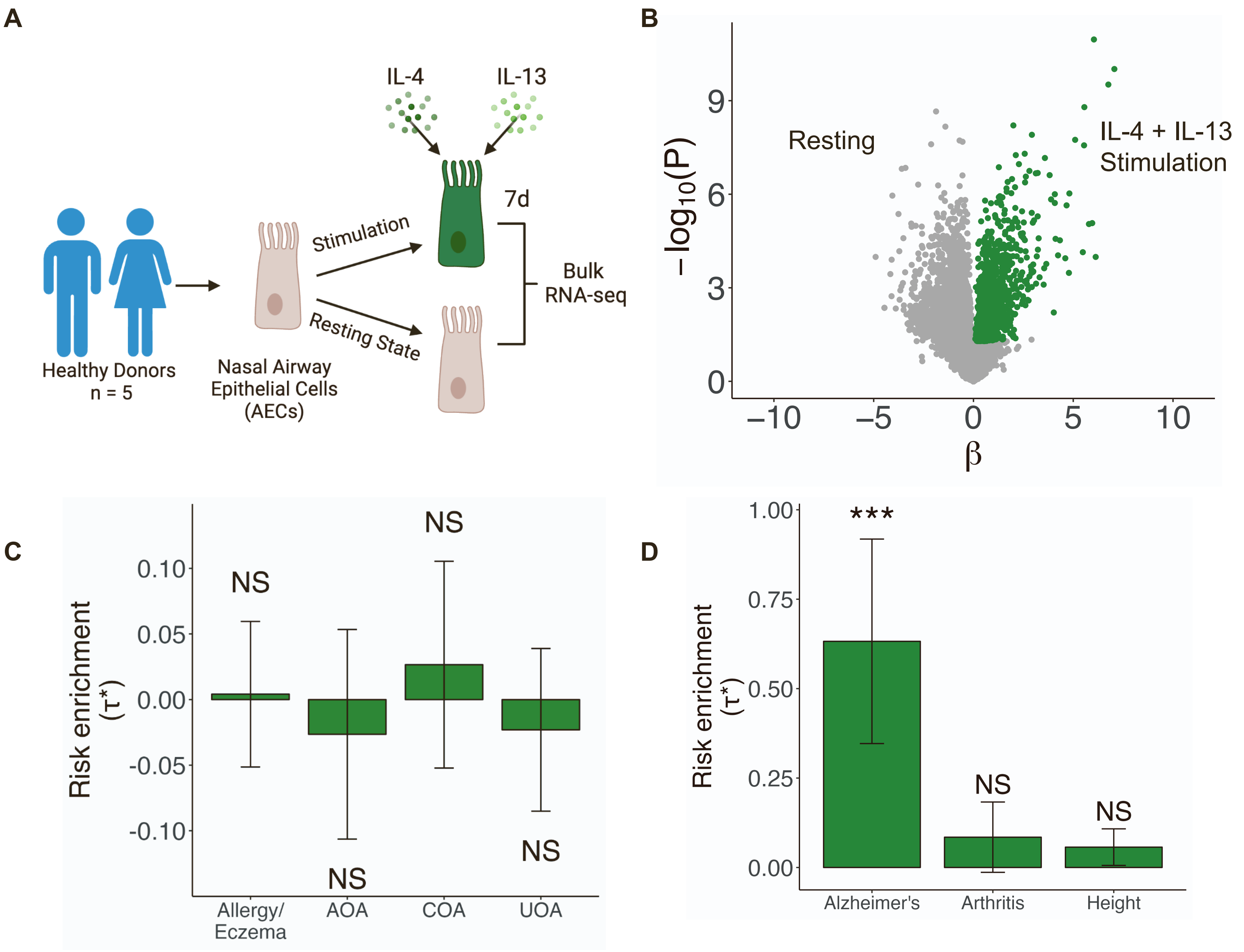

Supplementary Figure 13. Results for the analysis genes induced by cytokines (IL-4 and IL-13) in airway epithelial cells. Related to STAR methods. (A) Experimental design of bulk RNA-seq of airway epithelial cells from healthy donors co-stimulated with IL-4 and IL-13. **(B)** Volcano plot showing differentially expressed genes between IL-4/IL-13 stimulation and resting condition. Genes upregulated upon IL-4/IL-13 stimulation were selected based on t-statistic and are colored in green. **(C)** Bar plot showing LDSC-SEG enrichment for each of the asthma-related diseases. Error bars represent τ* +/- standard error. NS denotes nonsignificant ($P > 0.05$). **(D)** Bar plots representing LDSC-SEG heritability enrichment coefficient (τ*) for the 3 control traits. Error bars represent τ* +/- standard error. Asterisk denotes significance as * $P < 0.05$ and NS denotes nonsignificant ($P > 0.05$). AOA: Adult-Onset Asthma, COA: Childhood-Onset Asthma, UOA: Unspecified-Onset Asthma

Supplementary References

1. Jansen, I.E., Savage, J.E., Watanabe, K., Bryois, J., Williams, D.M., Steinberg, S., Sealock, J., Karlsson, I.K., Hägg, S., Athanasiu, L., et al. (2019). Genome-wide meta-analysis identifies new loci and functional pathways influencing Alzheimer's disease risk. Nat. Genet. *51*, 404–413.
